# Towards genomic medicine: a tailored next-generation sequencing panel for hydroxyurea pharmacogenomics in Tanzania

**DOI:** 10.1186/s12920-024-01924-5

**Published:** 2024-07-18

**Authors:** Siana Nkya, Collin Nzunda, Emmanuel Saukiwa, Frida Kaywanga, Eliud Buberwa, David Solomon, Heavenlight Christopher, Doreen Ngowi, Julieth Johansen, Florence Urio, Josephine Mgaya, Salman Karim, Mohamed Zahir Alimohamed, Raphael Z. Sangeda, Clara Chamba, Emile R. Chimusa, Enrico Novelli, Julie Makani

**Affiliations:** 1 Department of Haematology and Blood Transfusion, Dar es Salaam, Muhimbili University of Health and Allied Sciences, Dar es Salaam, Tanzania; 2 Department of Biochemistry and Molecular Biology, Muhimbili University of Health and Allied Sciences, Dar es Salaam, Tanzania; 3 Tanzania Human Genetics Organisation, Dar es Salaam, Tanzania; 4 Sickle Cell Program, Muhimbili University of Health and Allied Sciences, Dar es Salaam, Tanzania; 5 Department Pharmaceutical Microbiology, Muhimbili University of Health and Allied Sciences, Dar es Salaam, Tanzania; 6 Department of Applied Sciences, Faculty of Health and Life Sciences, Tyne and Wear, Northumbria University, Newcastle, NE1 8ST UK; 7 Vascular Medicine Institute, School of Medicine, University of Pittsburgh, Pittsburgh, USA; 8 Muhimbili University of Health and Allied Sciences, Dar es Salaam, Tanzania; 9 Imperial College London, Exhibition Rd, South Kensington, London, SW7 2BX UK

**Keywords:** Sickle cell disease, Pharmacogenomics, Genomic medicine, Sequencing, Miseq illumina platform, Targeted panel, Tanzania

## Abstract

**Background:**

Pharmacogenomics of hydroxyurea is an important aspect in the management of sickle cell disease (SCD), especially in the era of genomic medicine. Genetic variations in loci associated with HbF induction and drug metabolism are prime targets for hydroxyurea (HU) pharmacogenomics, as these can significantly impact the therapeutic efficacy and safety of HU in SCD patients.

**Methods:**

This study involved designing of a custom panel targeting *BCL11A, ARG2, HBB, HBG1, WAC, HBG2, HAO2, MYB, SAR1A, KLF10, CYP2C9, CYP2E1* and *NOS1* as potential HU pharmacogenomics targets. These genes were selected based on their known roles in HbF induction and HU metabolism. The panel was designed using the Illumina Design Studio (Illumina, San Diego, CA, USA) and achieved a total coverage of 96% of all genomic targets over a span of 51.6 kilobases (kb). This custom panel was then sequenced using the Illumina MiSeq platform to ensure high coverage and accuracy.

**Results:**

We are reporting a successfully designed Illumina (MiSeq) HU pharmacogenomics custom panel encompassing 51.6 kilobases. The designed panel achieved greater than 1000x amplicon coverage which is sufficient for genomic analysis.

**Conclusions:**

This study provides a valuable tool for research in HU pharmacogenomics, especially in Africa where SCD is highly prevalent, and personalized medicine approaches are crucial for improving patient outcomes. The custom-designed Illumina (MiSeq) panel, with its extensive coverage and high sequencing depth, provides a robust platform for studying genetic variations associated with HU response. This panel can contribute to the development of tailored therapeutic strategies, ultimately enhancing the management of SCD through more effective and safer use of hydroxyurea.

**Supplementary Information:**

The online version contains supplementary material available at 10.1186/s12920-024-01924-5.

## Background

To date, four therapeutic agents have been approved for the management of SCD; L-glutamine, crizanlizumab, voxelotor, and hydroxyurea. Hydroxyurea was first approved for use in SCD in 1998 [1]. Although the mode of action of hydroxyurea has not been completely elucidated, hydroxyurea induces gamma-globin expression that leads to increased HbF [2, 3]. The proposed mechanism underlying this effect is the inactivation by hydroxyurea of the enzyme ribonucleotide reductase [4] leading to the induction of stress erythropoiesis [5], which favours an increased production of F cells over red blood cells with haemoglobin S. Hydroxyurea stands out from the rest of the therapeutic agents that induce HbF due to its unique features such as easy oral administration and low toxicity [1].

Multiple hydroxyurea benefits are linked to the augmentation of fetal hemoglobin (HbF) levels, which impedes the polymerization of abnormal hemoglobin S, resulting in a reduction in painful episodes, hospitalization rates, acute chest syndrome occurrences, blood transfusion requirements, and mortality in SCD [6]. Hydroxyurea treatment is additionally associated with increased hemoglobin levels and mean red cell volume of red blood cells (RBCs), as well as decreased counts of white blood cells, platelets, and reticulocytes [7, 8].

To date, many SCD patients have received hydroxyurea treatment, and those who have responded well have experienced a major improvement in their quality of life. However, for up to 30% of these patients, the intervention does not lead to clinical improvement [9, 10]. Non-compliance to treatment, individual variability and genetic factors in sickle cell disease (SCD) patients have been associated with the lack of response to hydroxyurea [9]. Individual variability has been associated with variations in drug metabolism and clearance rates, differences in drug absorption and distribution within the body as well as variations in target receptor expression or sensitivity. On the other hand, genetic factors that influence with HU response are believed to be those associated with HbF induction and HU metabolism [5, 11]. Consequently, individuals with high levels of HbF prior to HU initiation are known to respond better to HU treatment than those with low levels [5]. Variations in HbF levels in both individuals with and without SCD have been associated with genetic variants within the β-globin gene (*HBB*) and the genes coding for *BCL11A, MYB, HBG, KLF1, GATA1, ZBTB7A, HOXA9, HBG2, CHD4, MBD3* and *LDB1 MAP3K5, ASS1, NOS2A, TOX, PDE7B, NOS1, FLT1 and ARG2* [12, 13]. As a result, these variants are also potential targets for pharmacogenomic approaches to predict response to HU. Among these, the study focused on investigating variants in *BCL11A, ARG2, HBB, HBG1, WAC, HBG2, HAO2, MYB, SAR1A, KLF10, CYP2C9, CYP2E1* and *NOS1* genes.

The selection of these genes was based on extensive literature review and their known roles in hydroxyurea response pathways. These genes play various roles related to hemoglobin synthesis, chromatin remodelling, transcriptional regulation, metabolism, and vascular function. Among them, *MYB* regulates haematopoiesis, *HBB, HBG1*, and *HBG2* are involved in hemoglobin synthesis, and *BCL11A* influences gamma-globin expression. *KLF10* acts as a transcriptional repressor(major silencer of HbF expression) [5], *HAO2* may impact HU metabolism, and *NOS1* is involved in nitric oxide synthesis affecting vascular function. *ARG2* influences arginine metabolism, *SAR1A* is associated with vesicular transport and gamma-globin expression, while *CYP2C9* and *CYP2E1* are involved in drug metabolism pathways.

Pharmacogenomic studies (aimed at identifying genes associated with a drug response) have proven important especially in (i) improving drug efficacy and safety, (ii) identifying serious side effects, (iii) predicting drug efficacy and enhancing genomic medicine. This field is also regarded as an innovative approach with a great potential of improving medicine both in developed and developing countries [14]. Pharmacogenomics tests are now implemented as part of clinical practice and in 2014, the U.S. Food and Drug Administration (FDA) released guidelines for this [15]. Although pharmacogenomics studies are increasingly common in high-income countries (HICs), their adoption and application in low- and middle-income countries (LMICs) have been relatively limited [16]. The situation is the same for pharmacogenomics studies for SCD treatments such as HU. This research gap is particularly significant in Africa due to the high prevalence and burden of sickle cell disease (SCD). It is crucial to promptly address this disparity in order to bridge the gap in pharmacogenomics studies and improve healthcare outcomes for individuals with SCD in Africa. This study aimed at reducing this disparity by outlining the methodology employed in a study on hydroxyurea pharmacogenomics conducted in Tanzania. Specifically, we describe the processes involved in the design and implementation of Illumina’s custom AmpliSeq™ next-generation sequencing panel, which focused on genes associated with hydroxyurea metabolism and HbF induction.

## Methods

### AmpliSeq panel-based designing

This study utilized a custom AmpliSeq panel from Illumina (Illumina, San Diego, CA, USA) for targeted sequencing. The panel was designed using the Illumina Design Studio (Illumina, San Diego, CA, USA) and achieved a total coverage of 96% of all genomic targets over a span of 51.6 kilobases (kb). Which means some regions will not be captured in the library preparation and in most cases, these correspond to high GC regions or regions with repetitive sequences. The panel was designed to capture the genomic regions of 13 specific genes, covering a total of 49,651 base pairs in the human genome. It consisted of 354 amplicons, and the primers were divided into two pools for library amplification as seen in Supplementary Table (1) The panel was designed to analyse genetic variants associated with HbF induction and HU metabolism as listed in Supplementary Table (2) This study also provides supplementary BED files containing the genomic positions of the missed bases in exons, as well as a summary of the exons containing missed bases in each gene (Supplementary Data 1).

### Sample collection and DNA extraction

The study included sixty (60) individuals aged five years (5yrs) and older who have not previously received hydroxyurea treatment and fulfilled specific haematological criteria for commencing hydroxyurea therapy including: HbA levels below 15%, absolute neutrophil count exceeding 2,000/uL, platelet count surpassing 100,000/uL, hemoglobin level greater than 5.0 g/dL, and absolute reticulocyte count over 100,000/uL at Muhimbili National Hospital (MNH), Amana, and Temeke regional hospital. A total of 4 ml of peripheral blood samples was collected from participants at baseline (prior to HU treatment initiation) and at each follow up visit. The DNA extraction process was manual and column-based using the QIAamp Blood Mini Kit (QIAGEN, Germantown, USA) and the buffers provided by the manufacturer (AL, AW1, AW2, EB, and proteinase K). The quantity of the extracted DNA was assessed using the Qubit DNA High Sensitivity (HS) Assay Kit with a Qubit 2.0 Fluorometer, while the quality of the DNA was evaluated using a NanoDrop™ 2000/2000c Spectrophotometers (ThermoFisher Scientific, Waltham, USA).

### Library preparation and sequencing

The extracted DNA was diluted first to 20 ng with confirmation of the concentration using a Qubit 2 fluorometer (Thermo Fisher Scientific) with a Qubit DNA broad-range assay kit (Thermo Fisher Scientific). Finally, samples were further diluted to 4 ng/µL prior to use. Five µL of each sample (20 ng) was then processed using an Ampliseq Library Plus for Illumina kit (Illumina, San Diego, CA, USA) together with the custom DNA panel. The amplified sample was partially digested and indexed using Illumina’s AmpliSeq CD indexes set A(Illumina). The library was then purified using AMPure XP beads (Beckman Coulter, Mississauga, Canada), amplified and recleaned prior to evaluation. Sample aliquots were used to determine the DNA concentration (ng/µL) using a Qubit 2 fluorometer instrument with a dsDNA HS assay (Thermo Fisher Scientific).

Library size distribution was checked by Agilent High Sensitivity DNA Kit on 2,100 Bioanalyzer (Agilent Technologies, Santa Clara, USA), from which a distinct peak between 350 and 400 bp should be observed, with a negligible level of contaminating DNA species. Based on these analyses, the sample’s nanomolar concentration was estimated and samples were normalised by dilution to a final concentration of 4 nM. The pooled library was then diluted to 10 pM and spiked with 5% 10 pM PhiX DNA as technical control. The pooled library was then loaded to the MiSeq sequencing cartridge v3 Kit with 2 × 300 bp with the 600-cycle and subject to massive parallel sequencing to generate 150 bp paired-end reads in Illumina MiSeq System.

### Data analysis

The MiSeq sequencing platform’s built-in software generated FASTQ output files from the raw reads, and quality control (QC) metrics were determined. The Sequence Analysis Viewer (SAV) app from Illumina and FastQC from the Bioinformatics Group at the Babraham Institute were used to monitor sequencing QC metrics for both the pooled library and individual libraries. To process the sequencing data, the DNA Amplicon software (v2.1.1: Illumina, San Diego, CA, USA), a commercial pipeline, was employed. This pipeline performed read alignment against the reference genome (GRCh38 p.2), filtering, and variant calling. The data analysis focused on the coding exons of the targeted genes. Additional custom filtering criteria were implemented to minimize false-positive rates. The generated mutation report in VCF format included annotations for all variants. To gain a deeper understanding of these variants and analyse the raw reads, various tools like ANNOVAR, Ensembl Variant Effect Predictor (VEP), and Integrative Genomics Viewer (IGV) [17] were employed.

## Results

### Sample collection, DNA extraction, library preparation and sequencing metrics

A total of 60 samples were used as part of piloting the designed panel. The quantity of DNA ranged from 300 ng/ul to 500 ng/ul which was sufficient for library preparation and sequencing. The A_260_/A_280_ ratio of the extracted DNA varied between 1.7 and 2.1, indicating the good range of DNA quality.

**Fig. 1:**
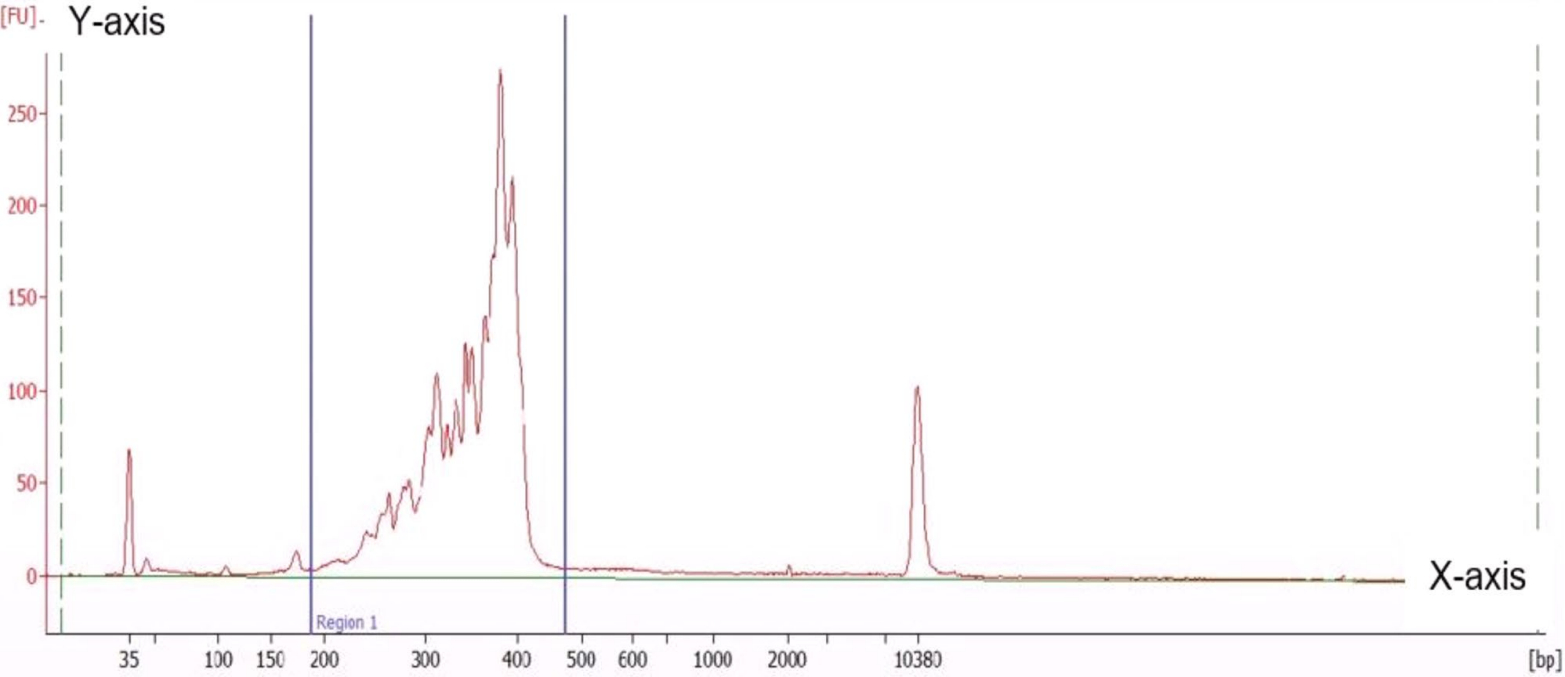
The sequencing library fragment analysis performed using the 2100 Agilent Bioanalyzer with the High Sensitivity DNA Chip. The figures visually represent the distribution of fragment sizes obtained from the analysis. The average fragment size recorded in this analysis was 380 bp

The libraries exhibited satisfactory yield and quality, as evidenced by concentration values ranging from 5 to 12 ng/µL and average sizes ranging from 350 to 390 bp as seen in Fig. 1. Notably, there was a clear and prominent peak observed between 300 and 400 bp. The total yield of each sequencing run ranged from 1.74 to 2.98 giga-bps and the cluster density ranged from 1000 to 1400 k/mm^2^. Overall, the sequencing quality was good, with %Q30 values ranging from 92 to 96%. The PhiX alignment rate was between 4.5% and 5.1%. Importantly, all individual libraries successfully passed the FastQC metrics, indicating high-quality sequencing data.

### Statistics of AmpliSeq sequencing for Illumina custom panel

Three runs were performed: three batches, each containing a different number of samples; batch 1 contained 4 samples, batch 2 contained 6 samples, and batch 3 contained 50 samples. The quality control (QC) parameters of sequencing using the AmpliSeq panel were acceptable for all runs. The specific values of QC parameters are listed in Table 1.

**Table 1:** This table shows the run statistics of sequencing using AmpliSeq for the Illumina custom panel from each batch

	Batch 1	Batch 2	Batch 3
Percent Q30 bases	95	96.23	93.7
Coverage at 100X	100.00	100.00	100.00
Uniformity of the base coverage at 0.2, %	86.16	92	90.4
Amplicon mean coverage	10995.2	10,463	1026.87
Percent on target aligned reads	98.5	98.1	90.7
Number of Amplicon Region	354	354	354
Total length of target regions	63,046	63,046	63,046
Percent aligned reads	87.1	89.9	87.6

### Coverage and variant summary of the custom ampliseq panel

**Fig. 2:**
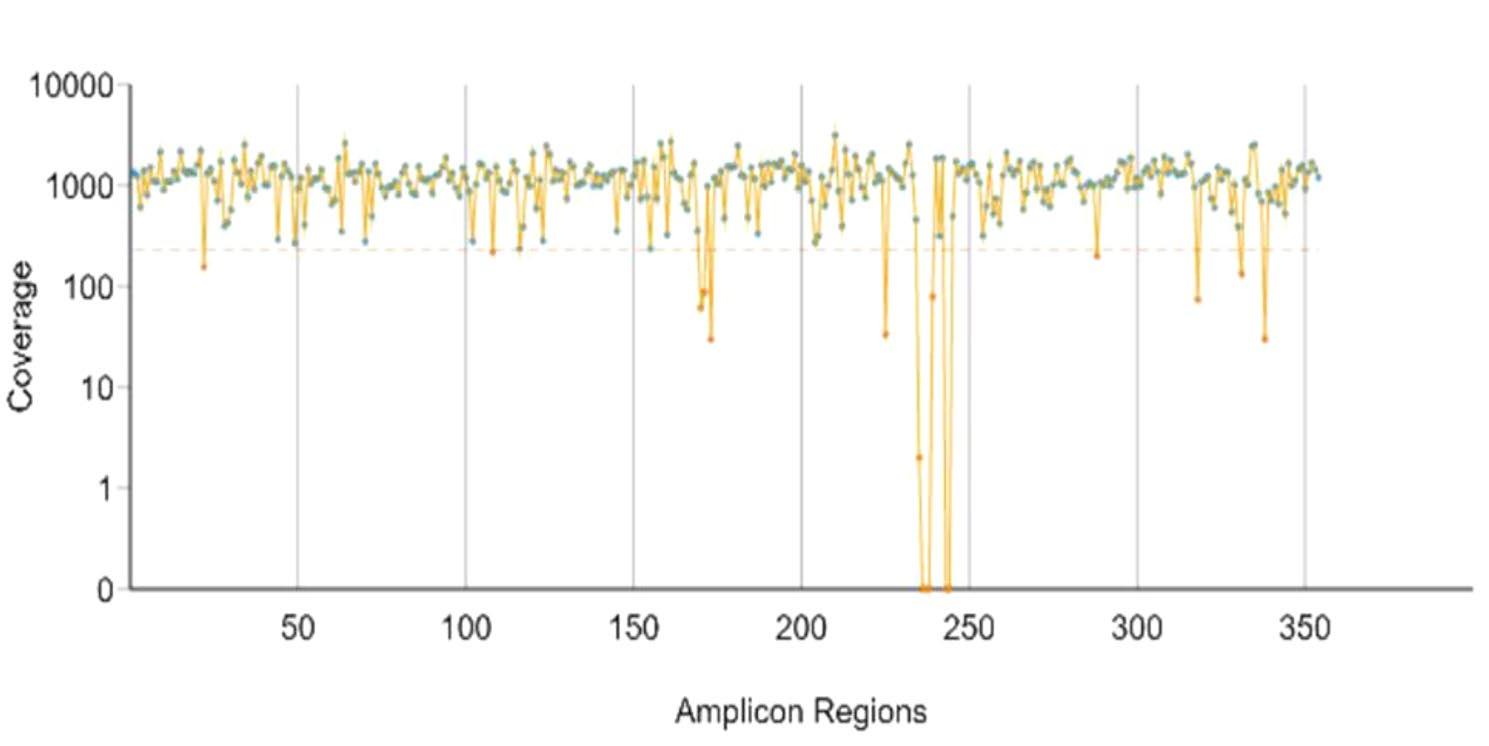
Shows coverage of the Amplicon region of one of the sequencing libraries of about 1156x, it had a uniformity of coverage (Pct > 0.2* mean) of about 95.2

**Fig. 3:**
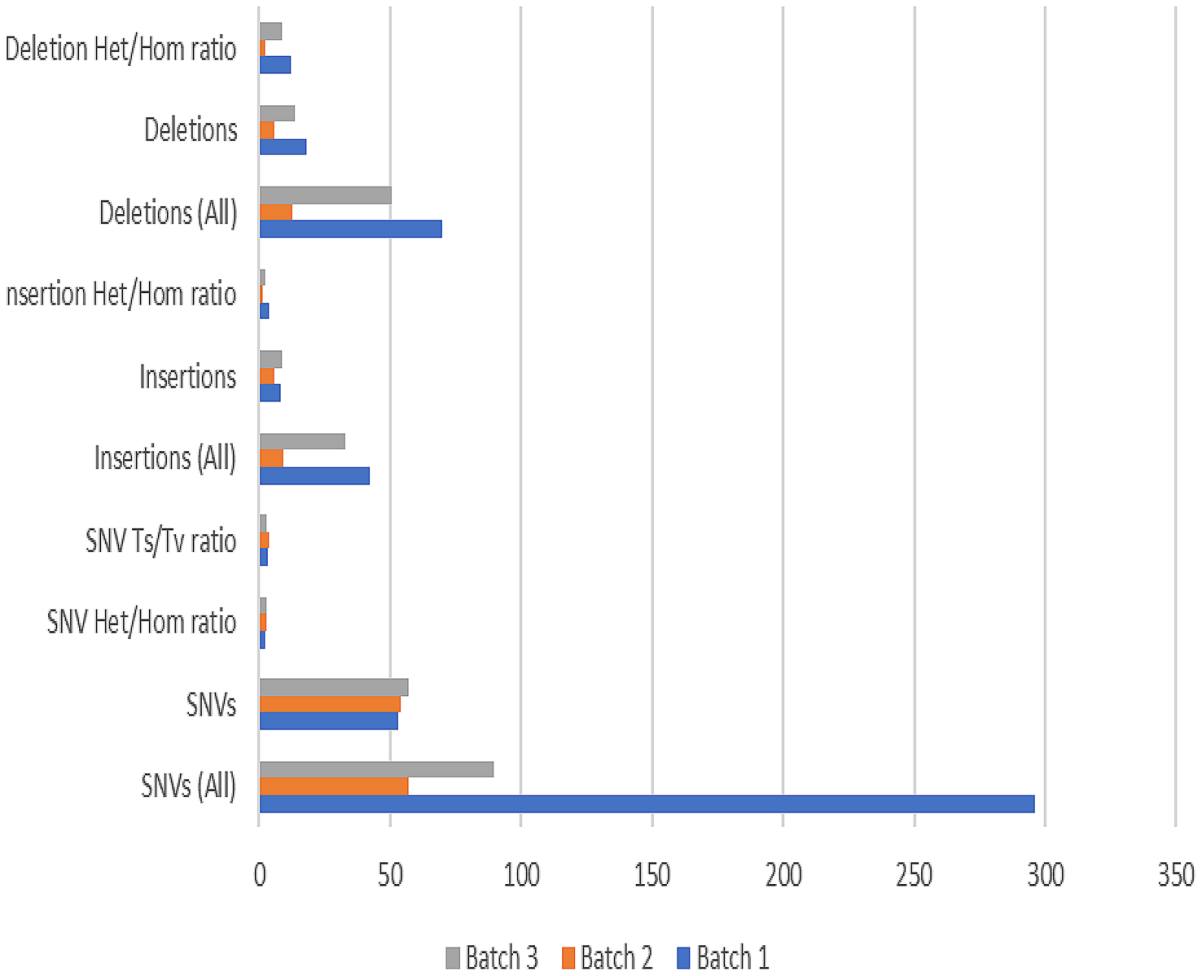
Summary of genetic variants detected in three batches

After alignment against the reference genome (GRCh38/hg38), on average one library had 3,892,305 reads on targets in batch 1, 3,703,961 reads on targets in batch 2 and 363,511 reads on targets in batch 3, with coverage of greater than 100×, as seen in Table 1; Fig. 2. For each of the 13 genes, we looked at the percentage of > 100× coverage in the reportable region from the three sequencing runs. The uniformity of the coverage (defined as % of base with > 0.2× mean coverage) was low in one library from batch 1 with 48.9% which will not be included in the downstream analysis. In the genetic variant analysis as seen in Fig. 3, three batches showed variations in the number and ratio of SNVs, insertions, and deletions.

Heterozygous variants were predominant, and the Ts/Tv ratio suggested a bias towards transition mutations. Variability across batches may be influenced by sample composition or experimental conditions.

## Discussion

Globally, different hydroxyurea pharmacogenomics studies have taken different approaches, including, whole exome and targeted sequencing focusing on different regions [18]. We chose a targeted approach in the selection of genomic regions to be analysed. The development of the panel in this study was based on the following; (i) hydroxyurea response is influenced by the levels of HbF an individual possesses before the initiation of treatment (baseline HbF levels), (ii) individual’s HbF level is significantly influenced by genetic factors associated and non-associated with SCD haplotypes. It is not clear how baseline HbF levels influence hydroxyurea response, however since HbF levels are genetically influenced then hydroxyurea response may have a direct or indirect association with genetic variants that influence HbF. It is also postulated that hydroxyurea response may be influenced by genetic variants associated with drug metabolism. However, it is not known whether there is an interaction between genetic variants associated with HbF with those influencing drug metabolism. This knowledge is important in order to increase understanding of hydroxyurea administration and genomic medicine as well as understanding how the mechanism of hydroxyurea response should be taken into consideration especially when administering hydroxyurea in combination with other drugs. Our study is novel as we have targeted genomic regions associated with HbF induction and hydroxyurea metabolism (Supplementary Table 2).

Similarly, we chose to perform sequencing using a targeted approach. Targeted next-generation sequencing (NGS) technologies provide powerful platforms for the detection of genetic variations, encompassing single nucleotide polymorphisms (SNPs), insertions, deletions, and other genomic variations. The design of a custom panel entails the careful selection of relevant genomic regions and optimization of coverage, considering factors such as sequencing depth and the utilization of appropriate software tools for panel design. The targeted sequencing strategy employed in this study demonstrated improved accuracy in genotyping common functional variants across exonic and intergenic regions in clinically relevant pharmacogenes compared to traditional genotyping or sequencing strategies [16]. Additionally, it facilitated the comprehensive identification of novel/rare single nucleotide variants (SNVs) with efficient and rapid performance. The use of bioinformatics tools further enabled the customized analysis of sequencing data at various scales, providing flexibility in data utilization.

The sequencing analysis of three batches revealed that the reads had high quality, with over 95% of bases having a Q30 score. The desired coverage at > 100x depth was achieved, ensuring sufficient reads for accurate variant detection, similar result was observed to a pharmacogenomic study which also utilized targeted next generation sequencing as a tool for genomic medicine [16]. In contrast to targeted sequencing, the Whole Exome Sequencing approach used in a similar study investigating hydroxyurea pharmacogenomics in SCD exhibited a slightly lower coverage, with an average depth greater than 20x. Despite this difference, the achieved a quality control rate of approximately 92%, indicating a high level of sequencing accuracy [19]. Targeted sequencing surpasses Whole Exome Sequencing by providing a higher coverage depth that ensures a more comprehensive & accurate analysis of specific regions of interest. The base coverage was relatively even across target regions, minimizing the risk of missing important genetic variants. Although there was variation in amplicon coverage between batches, a significant proportion of reads aligned to the intended target regions. Overall, the sequencing runs were successful and reliable for detecting genetic variants. The analysis of three batches revealed variations in the number and characteristics of genetic variants detected. Batch 1 had the highest total count of SNVs, insertions, and deletions, while Batch 2 had the lowest counts. The ratio of heterozygous to homozygous SNVs increased across the batches. The ratio of transitions to transversions remained consistent. These findings suggest that different batches may exhibit distinct genetic variant profiles, possibly due to variations in sample composition. It highlights the importance of considering batch-specific effects when interpreting and comparing genetic variant data.

This study is among the pioneering work conducted in Tanzania and Africa that document the groundbreaking efforts involved in developing and applying a sequencing panel for hydroxyurea (HU) pharmacogenomics. Hydroxyurea pharmacogenomics is increasingly important in countries with high burden of sickle cell disease (SCD) as we progress to the era of genomic medicine. This work makes an important contribution towards the development of a custom-designed Hydroxyurea pharmacogenomics panel for clinical use, which will be beneficial for improving healthcare. In addition, it provides a platform for research that will lead to identification of new targets for drug development or alternative interventions like gene therapy. Despite the high burden of SCD in Africa and increasing access to hydroxyurea treatment, there are limited pharmacogenomics studies [16].

Designing and optimizing a sequencing panel targeting genes associated with hydroxyurea metabolism and HbF change remains an important gateway in identifying genetic variants associated with hydroxyurea response in SCD patients and hence facilitating genomic medicine. However, identifying, and designing genetic variants associated with hydroxyurea metabolism requires a robust design taking into consideration many factors that are not always straightforward. From the three sequencing runs, batch 3 was more cost-effective since we were able to obtain enough coverage and good coverage uniformity in regard to the high number of samples included in the run.

## Conclusion

This study presents the initial endeavour in Tanzania to develop and analyse a hydroxyurea pharmacogenomics panel, offering significant insights to scientists and researchers, particularly in Africa where resource constraints are prevalent. In this study, we found a custom AmpliSeq panel revealed satisfactory analytic performance and proved to be a suitable tool for detecting genetic variants that have been associated with HU metabolism and HbF changes. However, limitations were identified in library preparation and panel optimization, highlighting the need for improvements to ensure optimal coverage across the targeted genes. Advances in methodologies and bioinformatics analysis are necessary to overcome these challenges. Overall, the study highlights the potential use of the custom AmpliSeq panel in implementing hydroxyurea pharmacogenomics studies.

### Electronic supplementary material

Below is the link to the electronic supplementary material.

Supplementary Material 1

Supplementary Material 2

## Data Availability

Genomic sequencing data generated in this study have been submitted to the NCBI BioProject database (https://www.ncbi.nlm.nih.gov/bioproject/) under accession number PRJNA1114738.

## Abbreviations

gDNA: Genomic DNA
Hb: Haemoglobin
HbF: Fetal haemoglobin
HPLC: High Performance Liquid Chromatography
HS: High Sensitivity
HU: Hydroxyurea
IGV: Integrative Genome Viewer
IRB: Institutional Review Board
MUHAS: Muhimbili University of Health and Allied Sciences
Ng: Nanogram
PCR: Polymerase Chain Reaction
QC: Quality Control
RT: Room Temperature
SAV: Sequence Analysis Viewer
SNV: Single Nucleotide Variant
SCD: Sickle Cell Disease
VEP: Variant Effect Predictor
