# Supplementary material for: Towards genomic medicine: a tailored next-generation sequencing panel for hydroxyurea pharmacogenomics in Tanzania
Source: BMC Med Genomics. 2024 Jul 18;17:190. doi: 10.1186/s12920-024-01924-5 (PMC11256457; doi:10.1186/s12920-024-01924-5)
Supplement: Supplementary file 1 — Supplementary Material 1 [file 12920_2024_1924_MOESM1_ESM.docx]

**Supplementary Table 1**: Custom panel designed using Illumina design studio sequencing (Illumina, San Diego, CA, USA), achieved a total coverage of 96% over a span of 51.6 kilobases (kb).

| **Assay version** | **Ampliseq DNA Gene** |
| --- | --- |
| Species | Homo Sapiens (GRCh38.p2) |
| Sample Type | Regular |
| No. of Amplicons | 354 |
| Total target size (bp) | 51583 |
| Covered Bases (bp) | 49651 |
| Number of Pools | 2 |
| Amplicon per Pool | 178/176 |
| Max. Amplicon length | 275 |
| Stringency | High |
| Exon padding | 25 |
| Concentration | 2X |
| Coverage | 96.25% |

| **Target Gene** | **Target Gene Name** | **Function** |
| --- | --- | --- |
| *MYB* | MYB proto-oncogene | Regulation of haematopoiesis |
| *HBB* | Hemoglobin subunit beta | Synthesis of beta globin in HbA |
| *HBG1* | Hemoglobin subunit gamma 1 | Synthesis of gamma globin of HbF |
| *HBG2* | Hemoglobin subunit gamma 2 | Synthesis of gamma globin of HbF |
| *BCL11A* | BAF chromatin remodelling complex subunit | Haematopoietic cell differentiation and silencing of gamma-globin expression in adults |
| *KLF10* | Kruppel like factor 10 | The encoded protein is a transcriptional repressor that acts as an effector of transforming growth factor beta signalling. |
| *HAO2* | Hydroxyacid oxidase 2 | The encoded protein localizes to the peroxisome has the highest activity toward the substrate 2-hydroxypalmitate |
| *NOS1* | Nitric oxide synthase 1 | Synthesize nitric oxide from L-arginine |
| *ARG2* | Arginase 2 | Arginase catalyses the hydrolysis of arginine to ornithine and urea |
| *SAR1A* | secretion associated Ras related GTPase 1A | Encodes a GTP-binding protein called SAR1A, has been reported to be associated with HBG expression |
| *CYP2C9* | Cytochrome P450 family 2 subfamily C member 9 | The cytochrome P450 proteins are monooxygenases which catalyse many reactions involved in drug metabolism and synthesis of cholesterol, steroids and other lipids |
| *CYP2E1* | Cytochrome P450 family 2 subfamily E member 1 | Catalyses many reactions involved in drug metabolism and synthesis of cholesterol, steroids and other lipids. |

**Supplementary Table 2*:*** Genomic regions targeted by the custom Illumina Ampliseq panel. The target gene includes genes involved in hydroxyurea metabolism *(CYP2C9 & CYP2E1)* and HbF induction *(MYB, HBB, HBG1, HBG2, BCLL1A, KLF10, HA02, NOS, ARG2 and SAR1A).*
